# Reduced Neutralization Efficacy against Omicron Variant after Third Boost of BNT162b2 Vaccine among Liver Transplant Recipients

**DOI:** 10.3390/v15010253

**Published:** 2023-01-16

**Authors:** Yana Davidov, Victoria Indenbaum, Michal Mandelboim, Keren Asraf, Tal Gonen, Keren Tsaraf, Oranit Cohen-Ezra, Mariya Likhter, Ital Nemet, Limor Kliker, Orna Mor, Ram Doolman, Carmit Cohen, Arnon Afek, Yitshak Kreiss, Gili Regev-Yochay, Yaniv Lustig, Ziv Ben-Ari

**Affiliations:** 1 Liver Diseases Center, Sheba Medical Center, Tel-Hashomer, Tel Aviv 52621, Israel; 2 Central Virology Laboratory, Ministry of Health, Tel-Hashomer, Tel Aviv 52621, Israel; 3 Sackler Faculty of Medicine, Tel-Aviv University, Tel-Aviv 52621, Israel; 4 The Dworman Automated Mega Laboratory, Sheba Medical Center, Tel-Hashomer, Tel Aviv 52621, Israel; 5 Infection Prevention & Control Unit, Sheba Medical Center, Tel-Hashomer, Tel Aviv 52621, Israel; 6 General Management, Sheba Medical Center, Tel-Hashomer, Tel Aviv 52621, Israel

**Keywords:** BNT162b2 vaccine, neutralizing antibody, third vaccine dose, liver transplant recipients, Omicron

## Abstract

The immune responses of liver transplant (LT) recipients after the third boost of the BNT162b2mRNA vaccine improved. This study evaluates the durability of the immune response of LT recipients after the third boost, its predictors, and the impact of emerging variants. The receptor-binding domain IgG was determined at median times of 22 (first test) and 133 days (second test) after the administration of the third boost. IgG antibody titers > 21.4 BAU/mL were defined as a positive response. The neutralization efficacies of the vaccine against the wild-type, Omicron, and Delta variants were compared in the first test. The 59 LT recipients were of a median age of 61 years (range 25–82); 53.5% were male. Following administration of the third dose, the positive immune response decreased from 81.4% to 76.3% between the first and second tests, respectively, (*p* < 0.0001). The multivariate analysis identified CNI monotherapy (*p* = 0.02) and hemoglobin > 12 g/dL (*p* = 0.02) as independent predictors of a maintained positive immune response 133 days after the third dose. The geometric mean titers of Omicron neutralization were significantly lower than the wild-type and Delta virus (21, 137, 128, respectively; *p* < 0.0001). The immune response after the third BNT162b2mRNA vaccine dose decreased significantly in LT recipients. Further studies are required to evaluate the efficacy of the fourth vaccine dose and the durability of the immune response.

## 1. Introduction

Two doses of the BNT162b2 mRNA vaccine against severe acute respiratory syndrome coronavirus 2 (SARS-CoV-2) are safe and effective in immunocompetent subjects [1,2,3]. However, the waning of the vaccine’s protection over time against symptomatic infection after two doses of the vaccine has been reported among the immunocompetent and, more so, immunocompromised individuals [4,5,6,7,8]. Breakthrough infections have correlated with the level of humoral response [9]. Solid organ transplant (SOT) recipients have shown suboptimal immune responses following two doses of the vaccine [10,11,12,13]. Administration of a third boost has improved the humoral and cellular immune responses among immunocompetent individuals [14] and SOT recipients, although primarily kidney transplanted recipients (KTR) were included [11]. We have reported improved humoral immune responses among liver transplant (LT) recipients following the third dose of the BNT162b2 mRNA vaccine from 56% before the third vaccine to 98% after the third vaccine [15]. However, the critical issue regarding the durability of the immune response after the third boost remains unknown.

The recent emergence of the SARS-CoV-2 mutants named the Delta (B.1.617.2) and Omicron (B.1.1.529) variants of concern (VOC) by the World Health Organization (WHO) threatens the effectiveness of the currently introduced vaccines [5,7,16]. The number of mutations in the Omicron variant spike protein is twice that of the Delta variant, which has spread in many countries worldwide [17,18]. Since November 2021, Omicron variants have rapidly become the dominant SARS-CoV-2 VOC globally. The Delta variant was the predominant VOC documented in Israel between August and the end of December 2021. Since January 2022, the Delta variant has been replaced by the Omicron [19,20] variant. The Omicron variant was associated with a higher incidence rate but a lower case fatality rate [21]. The incidence of SARS-CoV-2 infection ranged from 0.16/100,000 to 82.95/100,000 during the Delta period and from 0.03/100,000 to 440.88/100,000 during the Omicron period [21].

Several studies were conducted to evaluate the neutralizing ability of sera against the Omicron and Delta variants among immunocompetent patients vaccinated with two or three doses of different vaccines [7,16,22,23]. Nemet et al. reported improved neutralization efficiency (by a factor of 100) in 20 healthcare workers against the Omicron variant following the third dose compared to after the second dose; however, after three vaccine doses, neutralization against the Omicron variant was lower (by a factor of four) than that against the Delta variant [7].

A reduced immune response against the Omicron VOC among KTRs was recently reported by Jurdi et al. [24]. Among the 51 KTRs, the neutralizing response against Omicron slightly improved four weeks after the third dose of mRNA vaccines (BNT162b2 or mRNA-1273) from 0 to 12% (*p* = 0.012) prior to and after the third boost, respectively [24].

Our LT recipients showed slightly better immune response after two and three doses of mRNA vaccines, compared to other SOT recipients [15]. However, the durability of the immune response and its efficacy against the Omicron VOC among LT recipients is unknown.

Our aim is to prospectively assess the durability of the cellular and humoral immune response after the third dose of BNT162b2, its predictors, and its impact on the emerging VOC among LT recipients.

## 2. Materials and Methods

This study was conducted at the Sheba Medical Center between 5 August 2021 and 5 January 2022. The study population included 59 adult (age > 18 years) LT recipients, routinely followed at the Liver Diseases Center, and a control group of immunocompetent Sheba healthcare workers (HCW) who received the third BNT162b2 vaccine at least 21 days prior to recruitment for this study, who has not had past or active infection with SARS-CoV-2. The two control groups were matched to the LT recipients. The immune responses of the LT recipients 21 days after the third vaccine were compared with those of 20 age- and sex-matched HCWs (herein referred to as the first group). The immune responses of the LT recipients 4 months (133 days) after the third vaccination were compared to those of 67 HCWs matched for age, sex, and time between administration of the third vaccine dose and blood sample collections for serology (herein referred to as the second group) (Table 1).

Serum samples for quantification of IgG antibodies against the receptor-binding domain (RBD) of SARS-CoV-2 were collected from all subjects for at least 21 days (herein termed the first test) and at least 4 months after the third vaccination (herein termed the second test) (Table 1).

Anti-RBD IgG and NA antibody levels were determined with median times of 22 (IQR, 21–28) and 133 (IQR, 131–138) days after vaccination, respectively, for 59 LT recipients. However, T-cell evaluations were available for only 11 LT recipients.

Demographic, clinical, and laboratory data were extracted from the patients’ electronic records. Tacrolimus or everolimus trough levels and routine blood tests were performed four months after the third vaccine dose. Renal function was calculated using the Chronic Kidney Disease Epidemiology Collaboration (CKD-EPI) equation. Chronic kidney disease was defined as eGFR of less than 60 mL/min/1.73 m2 for a duration of more than 3 months.

This study was approved by the institutional review board of the Sheba Medical Center (8008-20-SMC. Signed informed consent was obtained from each patient included in this study.

### 2.1. Serology Assay

#### 2.1.1. Antibody Detection Testing

Blood samples were centrifuged at 4000 *g* for 4 min at room temperature. The serum was tested for IgG antibodies against SARS-COV-2 spike RBD using the commercial automatic chemiluminescent microparticle immunoassay SARS-CoV-2 IgG II Quant (Abbott, IL, USA) according to the manufacturer’s instructions.

A SARS-CoV-2 pseudo-virus (psSARS-2) neutralization assay was performed, as previously described [25], to detect SARS-CoV-2 neutralizing antibodies (NA) using a green fluorescent protein (GFP) reporter-based pseudo-typed virus with a vesicular stomatitis virus (VSV) backbone coated with the SARS-CoV-2 spike (S) protein, which was generously provided by Dr. Gert Zimmer (Institute of Virology and Immunology (IVI), Mittelhäusern, Switzerland). Sera that could not reduce viral replication by 50% from 1 to 8 dilution or below were considered non-neutralizing.

IgG antibody titers above 21.4 international binding antibody units per milliliter (BAU/mL) were defined as positive (responders), while anti-RBD IgG below 21.4 BAU/mL was defined as negative (non-responders). The responders whose anti-RBD IgG levels dropped below 21.4 BAU/mL were defined as those who failed to maintain an immune response. Patients who were still considered responders 4 months after receiving the third vaccine dose were defined as having a maintained immune response.

#### 2.1.2. T-Cell Response Testing

Fresh peripheral blood mononuclear cells (PBMCs) were used in all ELISpot assays using the ELISpot IFN-γ kit (AID). Briefly, fresh PBMCs (2 × 105 cells in 50 μL) were placed in duplicate wells and stimulated with 50 μL SARS-CoV-2 peptide pools (S-complete, Miltenyi Biotech) (2 μg/mL per peptide). Phytohemagglutinin (PHA) was used as a positive control, and 4Cell Nutri-T-medium was used as negative control. After 16–20 h at 37 °C, 5% CO_2_, and 95% humidity, cells were removed and secreted IFN-γ was detected by adding an alkaline phosphatase-conjugated secondary antibody for 2 h. The plates were developed using BCIP/NBT substrate according to the manufacturer’s instructions. The ELISpot plates were scanned using an AID ELISpot reader. Nonspecific background (mean SFU from the negative control wells) was subtracted from the experimental readings.

#### 2.1.3. Viral Isolation of the Wild-Type, Delta and Omicron Variants and SARS-CoV-2 Micro-Neutralization Assay

The wild-type virus and VOC were isolated from nasopharyngeal samples from SARS-CoV-2-positive individuals, which contained the wild-type sublineage B.1.1.50 (hCoV-19/Israel/CVL-45526-ngs/2020), Delta (B.1.617.2; hCoV-19/Israel/CVL-12804/2021), and B.1.1.529, Omicron, BA.1 (hCoV-19/Israel/CVL-n49814/2021) variants. Confluent VERO-E6 cells were incubated for one hour at 33 °C with 300 μL of nasopharyngeal samples, after which 5 mL of 2% FCS 3MEM-EAGLE medium was added. Upon CPE detection, supernatants were aliquoted and stored at −80 °C, as previously described [26]. VERO-E6 cells were seeded in sterile 96-well plates with 10% FCS MEM-EAGLE medium and incubated at 37 °C for 24 h. One hundred TCID50 of the wild-type, Delta and Omicron (BA.1) SARS-CoV-2 isolates were incubated with inactivated sera diluted from 1:8 to 1:16; 384 were placed in 96-well plates for 60 min at 33 °C. Virus–serum mixtures were placed over the VERO-E6 cells and incubated for five days at 33 °C, after which a gentian violet stain (1%) was applied to fix and stain the cell culture layer. The neutralizing dilution of each serum sample was determined by identifying the well with the highest serum dilution without an observable cytopathic effect. A dilution of 1:10 or above was considered neutralizing [26].

#### 2.1.4. Statistical Analysis

Continuous data were presented as medians, and the interquartile range (IQR) and categorical data were expressed as counts (percentages). Log-transformed antibody titers were used for statistical analysis. Titers of anti-RBD IgG, NA, and the IFN-γ-secretin T-cells per 10^6^ PBMC were calculated for all groups and are presented as geometric mean titers (GMT) with 95% confidence intervals (CI). LT recipients were paired with anti-RBD IgG titer data on days 22 and 133, and matched HCWs were grouped by their IgG antibody levels to positive and negative immune responses. Descriptive statistics were performed using a chi-squared analysis and Mann–Whitney U test for categorical and continuous data, respectively. Non-parametric Wilcoxon paired tests were conducted to compare the quantitative data. Patients were categorized according to their anti-RBD IgG titer to assess the predictors of maintaining the immune response after the third dose. A logistic regression analysis model was used to explore the factors associated with the maintenance of the immune response after the third dose. Covariates for the multivariate models were selected based on clinical judgment and variables that differed significantly between the groups. Correlations were estimated between anti-RBD IgG and NA titers and IFN-γ-secreting T-cell counts using the Spearman correlation test. Additionally, *p* < 0.05 was considered a statistically significant difference. All tests were two-sided. Statistical analyses were performed using SPSS (IBM SPSS Statistics, version 25, IBM Corp., Armonk, NY, USA, 2016). Scatter plots of the analyzed data were produced using GraphPad Prism version 9.2.0 for Windows (GraphPad Software, San Diego, CA, USA).

## 3. Results

### 3.1. Baseline Characteristics

The baseline demographics and clinical and laboratory characteristics of the 59 LT recipients are presented in Table 2.

The median patient age was 66 years (interquartile range (IQR) 54–70 years; range: 25–82 years), and 59.3% were male. The median time since the LT was seven years (IQR 4–16 years). Three patients (5.1%) underwent combined liver and kidney transplantations. The most common comorbidities were hypertension (53.4%), diabetes mellitus (43.1%), chronic kidney disease (59.3%), and dyslipidemia (53.4%). Calcineurin inhibitors (CNI) were the principal immunosuppressive agents, administered to 58 patients (52 tacrolimus and 6 cyclosporine). CNI monotherapy was administered to 29 patients (49.2%), while 28 patients (47.5%) received double immunosuppression (combination of CNI and mycophenolate mofetil (MMF)—14 patients; CNI and mTOR inhibitors (everolimus)—8 patients; CNI and prednisone—5 patients, mTOR inhibitors and prednisone—1 patient), and 2 (3.4%) patients received triple immunosuppression therapy (combination of CNI, MMF, and prednisone) (Table 2).

### 3.2. Comparison of Immune Response between Liver Transplant Recipients and Immunocompetent Controls

**Figure 1:**
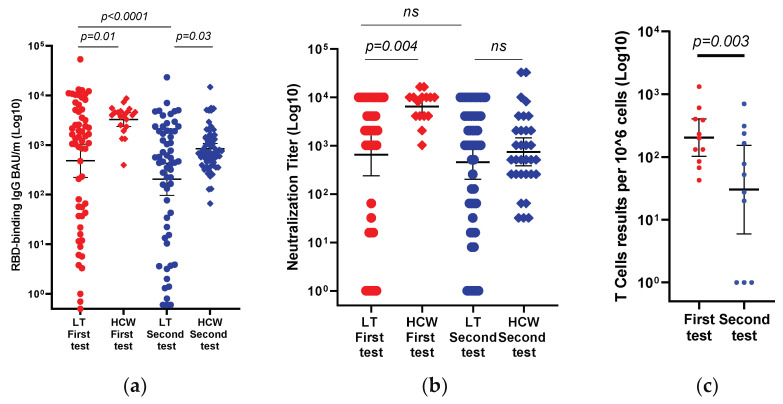
Comparison of immune responses of liver transplant recipients versus control immunocompetent subjects, after the third BNT162b2 vaccine dose was administered. Scatter plots showing the changes in anti-RBD IgG (**a**), neutralizing antibody (**b**) titers, and T-cell counts (**c**) at a median of 22 days (first test) and 133 days (second test) after administration of the third vaccine dose. The black horizontal line indicates the geometric mean values with a 95% confidence interval. Differences in paired samples were calculated using the Wilcoxon matched-pairs signed-rank test.

The immune responses among the LT recipients were compared to those of sex- and age-matched HCW. The initial immune response (first test) of the 59 LT recipients, assessed at a median time of 22 days (IQR, 21–28 days) following the third vaccine, was compared to that of a group of 20 sex- and age-matched HCWs at a median time of 33 days (IQR, 28–35 days) (Table 1). Compared to the HCW control group, the LT recipients showed a reduced immune response to the BNT162b2 vaccine (Table 1). A positive antibody response was documented in 48 of the 59 LT recipients (81.4%) compared to 20 of the 20 immunocompetent HCWs (100%) (OR 1.4 (CI 95% 1.2–1.7), *p* = 0.03) (Figure 1).

The geometric mean titers (GMT) of the IgG antibody and psSARS-2 NA in the LT recipients were lower than those in the HCWs (483 BAU/mL (95% CI, 225–1038) vs. 3297 BAU/mL (95% CI, 2373–4681), *p* = 0.01, and 653 (95% CI, 238–1795) vs. 6420 (95% CI, 4173–9879), *p* = 0.004, respectively) (Table 1, Figure 1).

The durability of the immune response (second time point) among the LT recipients was also compared between the 59 LT recipients and 67 HCWs who provided a blood sample 133 days after the third vaccination. A comparison of the demographic and laboratory characteristics of the two cohorts is presented in Table 1. The immune response among the LT recipients was significantly lower than that of the HCWs. A positive immune response was maintained in 45 of 59 (76.3%) of the LT recipients as compared to 67 of 67 (100%) of the HCWs [OR 2.5 (CI 95% 2.0–3.1), *p* < 0.0001]. The GMT of the IgG antibodies in the LT recipients was also significantly lower than that in the HCW (205 BAU/mL (97–433) vs. 844 BAU/mL (663–1074), respectively, *p* = 0.03). However, the geometric mean titers of psSARS-2 NA were similar in both groups (459 (201–1049) vs. 745 (385–1440), *p* = 0.8; Table 1, Figure 1).

### 3.3. Predictors of a Humoral Immune Response to THIRD BNT162b2 Vaccine Dose among LT Recipients

Following the univariate analysis, the LT recipients who did not develop anti-RBD IgG in the first test were older age at transplantation (60 years vs. 51 years, *p* = 0.02) and more likely to have chronic kidney injury (68.8% vs. 18.2%, *p* = 0.003). They had lower levels of their white blood cell counts (*p* = 0.03), hemoglobin (*p* = 0.003), chronic kidney injury (*p* = 0.003), and alanine aminotransferase (*p* = 0.0003) than the responders. A negative immune response was documented in patients who were treated with a lower dose of tacrolimus (*p* = 0.03), who were treated with MMF (*p* = 0.003), or who received combined (double or triple) immunosuppression (*p* = 0.006) (Table 2).

Independent predictors of a positive immune response 22 days after the third vaccine dose were CNI monotherapy (RR 12, CI 95%, 1.2–111, *p* = 0.04), a higher level of hemoglobin (RR 1.5, CI 95%, 1.0–2.4, *p* = 0.049), and absence of chronic kidney injury (RR 7, CI 95%, 1.1–46, *p* = 0.04). The age at vaccination, sex, time after transplantation, and comorbidities had no influence on the positive immune response that developed 22 days after the third dose.

A maintained immune response (second time point) was observed in 45 of 59 LT recipients (76.3%). The demographics and clinical and laboratory characteristics of the LT recipients who maintained an immune response after 133 days are presented in Table 3.

The independent predictors of maintaining the immune response for 133 days after the third vaccine were CNI monotherapy (RR 7.4 (CI 95%, 1.4–40.2, *p* = 0.02), younger age at transplantation (RR 0.9 (CI 95%, 0.8–0.99, *p* = 0.02), a higher hemoglobin level (RR 1.5, CI 95%, 1.1–2.2, *p* = 0.03), and a higher level of alanine transaminase (RR 1.2, CI 95%, 1.0–1.3, *p* = 0.02). The age at vaccination, sex, time after transplantation, and comorbidities had no influence on immune response maintenance after the third vaccine dose (Table 2).

The GMT of the anti-RBD IgG in the first test was 483 BAU/mL (CI 95%, 225–1038) and decreased significantly to 205 BAU/mL (97–433) in the second test (*p* <0.0001; Figure 1)). The GMT of the psSARS-2 NA titers between the first and second tests decreased from 653 (238–1795) to 459 (201–1049) BAU/mL, *p* = 0.2 (Figure 1).

The anti-RBD IgG titers were positively correlated with the psSARS-2 NA titers in both the first (r = 0.9, *p* < 0.0001) and second (r = 0.9, *p* < 0.0001) tests.

### 3.4. T-Cell Immunity to the Third Dose of the BNT162b2 Vaccine

In 11 LT recipients, the T-cell response was evaluated at medians of 22 and 133 days after the administration of the third vaccine dose. All patients had a positive T-cell response in the first test, while 3 of the 11 patients had a negative T-cell response in the second test. The GMT of the IFN-γ-secreting T-cells per 1 × 10^6^ PBMC decreased significantly between the first and second tests performed after the third dose (204 (95%CI, 103–404, range 43–1320) versus 30 (95%CI, 6–154, range 0–705), respectively (*p* = 0.003) (see Figure 1).

### 3.5. Neutralization of Wild-Type, Delta, and Omicron Variants 22 Days after the Third Vaccine Dose

**Figure 2:**
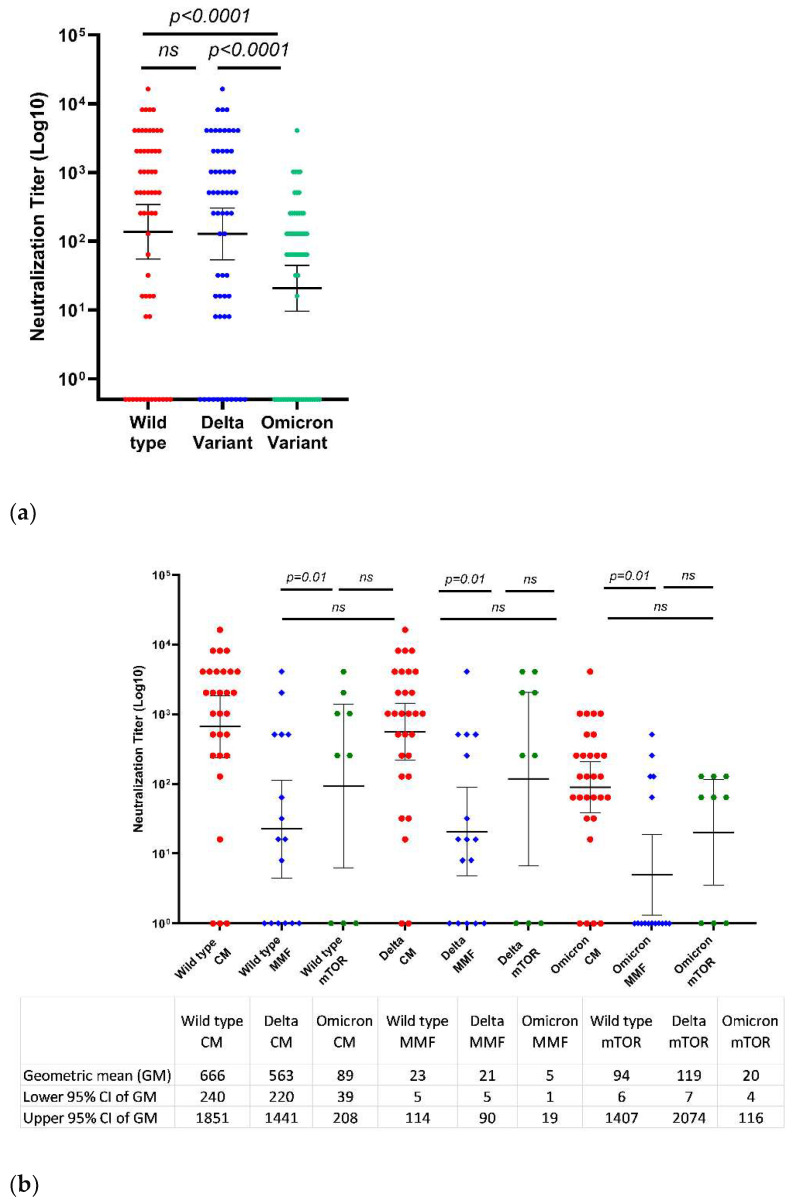

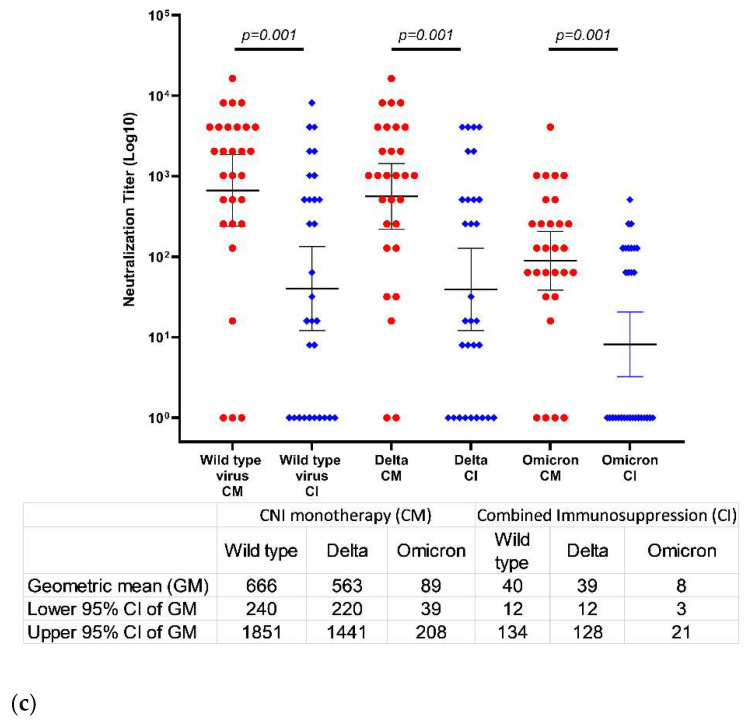
Neutralization of the wild-type SARS-CoV-2 virus and its Delta and Omicron variants of concern 22 days after the third BNT162b2 vaccine dose was administered. Scatter plots showing the neutralization efficacy of liver transplant patients against the wild-type SARS-CoV-2 virus and its Delta and Omicron variants (**a**), the influence of combined immunosuppression (CI) vs. CNI monotherapy (CM) (**b**), and the influence of MMF and mTOR inhibitors vs. CNI monotherapy (**c**). Differences in paired samples were calculated using the Wilcoxon matched-pair signed-rank test.

The effectiveness of the neutralizing antibodies against the wild-type virus and VOC (Delta and Omicron) was tested at a median time of 22 days after the third vaccine dose (first test). The third vaccine dose led to better neutralization of the wild-type and the Delta variants than of the Omicron variant (*p* < 0.0001) (Figure 2).

The GMT of the wild-type, Delta, and Omicron variants were 137 (55–343), 128 (54–305), and 21 (10–45), respectively.

Evaluation of the impact of the different types of immunosuppression therapy on the neutralization efficacy of the different types of SARS-CoV-2 variants following the third vaccine dose revealed that the GMTs of the wild-type, Delta, and Omicron variants were significantly higher among the LT recipients receiving CNI monotherapy than among those receiving combined immunosuppression (666, 563, 89 and 40, 39, 8, respectively, (*p* = 0.01) (Figure 2)). The neutralization efficacy was significantly lower among patients receiving the combination of CNI and MMF as compared to those receiving CNI monotherapy (Figure 2) (*p* = 0.01). Although the titer of neutralizing antibodies against the variants was numerically lower among the LT recipients receiving CNI in combination with MMF compared to mTOR in combination with CNI, and it was lower among those receiving CNI in combination with mTOR compared to CNI monotherapy, these differences did not reach statistical significance (Figure 2).

The anti-RBD IgG titers 22 days after the third vaccine correlated positively with their neutralization efficacy against the wild-type (r = 0.9, *p* < 0.0001), Delta (r = 0.9, *p* < 0.0001), and Omicron (r = 0.9, *p* < 0.0001) variants.

### 3.6. Breakthrough Infection after the Third Vaccine Dose

In a median follow-up period of 150 days (IQR,132–150 days) after the third vaccine, one patient developed the SARS-CoV-2 infection 120 days after the third dose. The only presenting symptom was a change in taste. A negative humoral immune response was documented 133 days after the third vaccine.

## 4. Discussion

Our study aimed to evaluate the waning rate of the immune response after the third vaccination among the LT recipients, compare it to the immune response among immunocompetent HCWs, and evaluate the impact of VOC on the neutralizing efficacy of the third vaccine among LT respondents. Four months after receiving the third vaccine dose, the humoral and cellular immune responses rapidly decreased among the LT recipients. Between days 22 and 133 post-vaccination, the positive humoral response, assessed by the levels of anti–RBD IgG, decreased from 81.4% to 76.3%. While the anti-RBD IgG titers and T-cell counts declined significantly, the level of psSARS-2 neutralizing antibodies remained relatively stable. The ability to maintain an immune response four months after the third vaccine was better among patients receiving CNI monotherapy than among those receiving combined immunosuppression therapy. Four months after vaccination, the immune response of the LT recipients was significantly impaired compared to that of age-, sex- and time after vaccination-matched immunocompetent HCWs, which was maintained in 100% of the HCWs.

Previous research has reported that after receiving the second vaccine dose, immune responses decline rapidly among transplant recipients but were significantly improved with the third dose [10,11,12]. Earlier studies also found that the type of immunosuppression used affected the immune response among LT recipients after the second vaccine [10,13]. Moreover, this projects the ability to maintain the immune response prior to the third vaccine [15]. A negative influence of MMF on the immune response among other SOTs was also reported after the third and second vaccines [10,11,12,13,15,27,28]. Kantauskaite et al. reported a dose-dependent effect of MMF on the immune response among KTRs [29]. KTRs receiving less than 1 g MMF daily had a better immune response to mRNA vaccines (OR 5.19, 95% CI 1.49–18.00, *p*= 0.009) [29].

Evidence of the transmission of the viral VOCs, which may escape the control induced by the vaccine even with the improved immune response reported after the booster vaccine, is accumulating [7,28]. Our results regarding the low immune response to the viral variants among the LT recipients are a matter of concern. We detected that the immune response to the wild-type, as well as to the Delta and the Omicron variants among LT recipients, was associated with impaired neutralization efficacy. The type of immunosuppression therapy administered by the patient significantly influenced the neutralization efficacy of the viral variants. The GMTs were lower among patients receiving CNI monotherapy than among those receiving a combination of mTOR with CNI or MMF with CNI. However, significant differences were only noted when comparing CNI monotherapy versus combined immunosuppression (combination of mTOR with CNI, MMF with CNI, and prednisone with CNI) and CNI monotherapy versus the combination of MMF and CNI. The lack of a significant correlation between the immune response and the combination of CNI with mTOR or CNI with prednisone is most probably related to the small number of LT recipients in these categories. The neutralization efficacy against VOCs is highly predictive of immune protection and provides an evidence-based model of SARS-CoV-2 immune protection [28]; however, the test is time-consuming and requires a biological safety level three facility and highly trained personnel. We showed a strong correlation between the anti-RBD IgG and neutralizing antibody titers. These findings may improve our diagnostic capability to predict immune responses with clinical implications in everyday practice.

Among the immunocompetent HCWs, the immune response to viral VOCs improved significantly after the third dose [7]. Moreover, it improved significantly after the fourth boost of both mRNA vaccines (BNT162b2 and mRNA-1273); however, its efficacy against infections was low [30]. Immunocompromised patients were not included in that study [30].

Due to the arrival of new VOC and the low effectiveness of the mRNA vaccines against the Omicron variants [31], the new bivalent mRNA containing the original SARS-CoV-2 strains and Omicron BA.4 and BA.5 sublineages was developed [32]. Since September 2022 a single boost of the bivalent mRNA vaccine has been recommended by the CDC. A recently published study showed the increased efficacy of the bivalent booster against COVID-19 in an emergency department/urgent care encounter or hospitalization compared to no vaccination in immunocompetent adults [33]. A similar study from Israel revealed the effectiveness of the bivalent vaccine among adult patients aged 65 years and older by demonstrating a decreased hospitalization and death rate due to COVID-19 [34].

The presented study was limited by its small sample size. The strength of this study lies in its being the first to evaluate the durability of the immune response after the third vaccine dose, and the first to evaluate the immune response to viral VOCs among LT recipients. It emphasized the important effect of the type of immunosuppression therapy on immune responses after vaccination with mRNA vaccines and its significant negative effect on neutralization efficacy against VOCs, mainly Omicron.

Future studies should address the effectiveness and durability of the newly developed bivalent vaccines against the Omicron variant among immunocompetent subjects and solid organ transplant recipients.

## 5. Conclusions

In conclusion, we present here a follow-up on the immune response four months after the third vaccine dose among LT recipients. The immune response decreased significantly compared to that in immunocompetent subjects. We detected a weak immune response 3 weeks after the third vaccine against the Omicron variant, which was almost null among the LT recipients who received combined immunosuppression. Further studies are required to evaluate the efficacy of the fourth vaccine dose on the durability of the immune response and protection against symptomatic COVID-19 disease.

## Figures and Tables

**Table 1:** Demographic characteristics and immunologic responses of liver transplant (LT) patients and healthy healthcare workers (HCW) to a third BNT162b2 mRNA vaccine dose.

Characteristics	First Test			Second Test		
	LT N = 59	HCW N = 20	*p* Value	LT N = 59	HCW N = 67	*p* Value
Age (years)	66 (54–70)	63 (51–67)	0.2	66 (54–70)	63 (49–66)	0.06
Male, *n* (%)	35 (59.3)	8 (40)	0.1	35 (59.3)	37 (55.2)	0.6
Time from third dose of the vaccine to serology testing, (days)	22 (21–28)	33 (28–35)	0.004	133 (131–138)	133 (129–138)	0.9
Positive IgG-RBD, *n* (%) ^§^	48 (81.4)	20 (100)	0.03	45 (76.3)	67 (100)	<0.0001
SARS-CoV-2 IgG titers, GMT (CI 95%), BAU/mL	483 (225–1038)	3297 (2373–4681)	0.01	205 (97–433)	844 (663–1074)	0.03
SARS-CoV-2 neutralizing antibodies, GMT (CI 95%)	653 (238–1795)	6420 (4173–9879)	0.004	459 (201–1049)	745 (385–1440)	0.8

Data presented as median (IQR) or *n* (%); § patients with IgG antibody titers above 21.4 BAU/mL were defined as a positive response; neutralizing antibody data in the control group were available for 32 patients only.

**Table 2:** Baseline demographics and clinical and laboratory characteristics of liver transplant (LT) patients with versus without immunologic response 22 days after the third BNT162b2 mRNA vaccine dose.

Characteristics	Total LT Cohort N = 59	Non-Responders ^§^ N = 11 (18.6%)	Responders ^§^ N = 48 (81.4%)	*p* Value
Age, years	66 (54–70)	66 (61–75)	64 (53–70)	0.2
Male	35 (59.3)	7 (63.6)	28 (58.3)	0.7
Indication for LT				0.3
Hepatitis C	13 (22)	4 (36.4)	9 (18.8)	
NASH	15 (25.4)	4 (36.4)	11 (22.9)	
Hepatitis B	4 (6.8)	0	4 (8.3)	
PSC	4 (6.8)	0	4 (8.3)	
PBC	4 (6.8)	1 (9.1)	3 (6.3)	
Other ¶	19 (32.2)	2 (18.2)	17 (35.4)	
Age at transplantation, years	53 (42–63)	60 (54–65)	51 (41–62)	0.02
Time since liver transplantation, years	7 (4–16)	6 (1–10)	9 (4–19)	0.07
Comorbidities				
Diabetes mellitus	25 (43.1)	7 (63.6)	18 (38.3)	0.1
Hypertension	31 (53.4)	8 (72.7)	23 (48.9)	0.1
Dyslipidemia	31 (53.4)	8 (72.7)	23 (48.9)	0.1
Chronic kidney disease	35 (59.3)	9 (81.8)	15 (31.3)	0.003
BMI, kg/m^2^	26 (22–28)	26 (23–27)	26 (22–28)	0.8
WBC, K/microL	5.6 (4.1–6.3)	4.7 (3.9–5.8)	5.8 (4.7–7.1)	0.03
Hemoglobin, g/dL	13.1 (11.7–14.5)	10.4 (9–11.7)	13.5 (12.8–14.6)	0.003
Platelets, K/microL	164 (125–197)	168 (130–241)	163 (124–188)	0.4
Creatinine, mg/dL	1.0 (0.8–1.3)	1.4 (1.2–2)	1 (0.8–1.2)	0.002
ALT, IU/L	20 (15–26)	12 (7–18)	21 (16–31)	0.003
ALP, IU/L	95 (74–128)	85 (72–106)	99 (77–130)	0.3
Bilirubin, mg/dL	0.6 (0.5–0.9)	0.5 (0.4–0.7)	0.6 (0.6–0.9)	0.07
Tacrolimus dose, mg/ trough level, μg/L	2.5 (1.5–4.0)/5.2 (4.1–6.3)	2 (1.5–4)/3 (2.9–5.4)	3 (1.5–4)/5.3 (4.5–6.6)	0.03/0.5
Prednisone, *n* (%)/dose, mg	8 (13.6)/10 (5–10)	2 (18.2)/5 (5–10)	6 (12.5)/10 (5–10)	0.6/0.4
MMF, *n* (%)/dose mg	16 (27.1)/875 (500–1000)	6 (54.5)/625 (500–1000)	10 (20.8)/1000 (500–1000)	0.03/0.4
Everolimus, *n* (%)/dose mg/trough level, ng/ml	9 (15.3)/2 (1.5–2.5)/2.9 (2.1–3.8)	3 (27.3)/4.2 (1.2–5.6)/2.5 (1.5–2.5)	6 (12.5)/2.8 (2.1–3.1)/1.8 (1.5–2.0)	0.2/0.4/0.6
Double ^‡^/triple ^‡‡^ immunosuppression	28 (47.5)/2 (3.4)	10 (90.9)/0	18 (37.5)/2 (4.2)	0.003

Data are presented as median (IQR) or *n* (%). § Patients with IgG antibody titers > 21.4 BAU/mL were defined as responders. Patients with titers < 21.4 BAU/mL were defined as non-responders. ‡ Double immunosuppression denotes combination of CNI and MMF—14 patients; CNI and everolimus—8 patients; CNI and prednisone—5 patients; mTOR inhibitors and prednisone—1 patient; ‡‡ Triple immunosuppression therapy was administered to 2 patients (combination of CNI, MMF, and prednisone). ¶ Other indications for liver transplantation: alcoholic liver disease, biliary atresia, cystic fibrosis, fulminant liver failure, Budd–Chiari syndrome. ALT, alanine aminotransferase; ALP, alkaline phosphatase; BMI, body mass index; LT, liver transplantation; MMF, mycophenolate mofetil; NASH, non-alcoholic steatohepatitis; PBC, primary biliary cholangitis; PSC, primary sclerosing cholangitis.

**Table 3:** Predictors of a maintained positive immune response 133 days after the third BNT162b2 vaccine dose.

Characteristics	Maintained Immune Response * N = 45 (76.3%)	No Maintained Immune Response * N = 14 (23.7%)	Univariate		Multivariate ^§^	
			OR (95% CI)	*p*-Value	OR (95% CI)	*p*-Value
**Age at transplantation, years**	51 (41–62)	59 (55–65)		0.004	0.9 (0.8–0.99)	0.02
**Time since liver transplantation, years**	9 (5–19)	6 (1–10)		0.04	1.1 (0.9–1.2)	0.08
**Chronic kidney disease**	15 (33.3)	9 (64.3)	3.6 (1.0–13)	0.04	2.1 (0.5–9.3)	0.3
**WBC, K/microL**	5.7 (4.7–7)	4.8 (3.9–6.2)		0.09	1.1 (0.8–1.6)	0.6
**Hemoglobin, g/dL**	13.6 (12.8–14.7)	11.1 (9.9–12.4)		0.003	1.5 (1.1–2.2)	0.03
**Hemoglobin > 12 g/dL**	39 (86.7)	4 (28.6)	16.2 (3.8–69)	<0.0001	13 (2.4–68)	0.003
**Creatinine, mg/dL**	1.0 (0.8–1.2)	1.3 (1.1–1.9)		0.01	0.9 (0.5–1.5)	0.7
**ALT, IU/L**	21 (16–31)	12.5 (8–18)		0.0001	1.2 (1.0–1.3)	0.02
**ALT > 15.5 IU/mL**	36 (80)	4 (28.6)	10 (2.5–39)	<0.0001	8.6 (1.8–40)	0.007
**CNI monotherapy**	27 (60)	2 (14.3)	0.1 (0.02–0.6)	0.003	7.4 (1.4–40.2)	0.02

* Patients with titer anti-RBD IgG levels > 21.4 BAU/mL 4 months after the third vaccine dose were defined as maintained immune response; patients with titers < 21.4 BAU/mL were defined as no maintained immune response. § A multivariable regression analysis of several models was performed using logistic regression analysis of CNI monotherapy, chronic kidney disease, or creatinine levels in combination with one of the following variables: age at transplantation, time since liver transplantation, white blood cells, hemoglobin, creatinine, and alanine transaminase levels. A receiver operator characteristic (ROC) analysis was performed to evaluate the accuracy of prediction of a maintained positive immune response. The area under the curve (AUC) for association of HB level to predict maintenance of a positive immune response 133 days after the third vaccine was 0.77, *p* = 0.003. HB level of 12 g/dL predicted positive immune response with sensitivity of 86% and specificity of 71%. The AUC for ALT was 0.82 (*p* < 0.0001). ALT > 15.5 IU/mL predicted positive immune response with sensitivity of 80% and specificity of 71%. Abbreviation: ALT, alanine transaminase; CNI, calcineurin inhibitors; WBC, white blood cells.

## Data Availability

Not applicable.
